# A Special Issue: Pharmaconosy

**Published:** 2017

**Authors:** Faraz Mojab


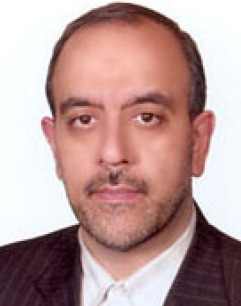


One of the special features of IJPR is the publication of special topic issues. This special issue is dedicated exclusively to articles about the field of Pharmacognosy. Pharmacognosy that means the science of medicine has a long history in Iran. Despite the fact that the word was first used in German literature about 200 years ago, its history dates back to 1,000 years ago in Iranian traditional medicine. The documents recorded by the Iranian ancient pioneer physicians, who made important contributions to the field, such as Rhazes, Avicenna, Jorjani, etc. have comprehensively referred to the principles of this science. Due to the variability of climate and geography, vegetation is very rich and strong in Iran. Thus, pharmacognosy research status in this country (and the world) is on the rise and the number of articles and documents obtained from this research is on the increase.

In this issue, several articles from authors in various fields of pharmacognosy have been offered. The result of these researches highlight the importance of antioxidant and cytotoxicity of plants (such as marine natural products), pharmacological activities on animal models, bioassay-guided studies, evaluation of plant effect used in Iranian traditional medicine, clinical trials, the analysis of essential oils, phytochemistry, antibacterial activity, various biological effects, etc. All of these areas may potentially lead the pharmaceutical and medical world to new natural sources for future drug discovery. 

In addition, IJPR special issues represent outstanding articles by scientists in the field of natural products research. This issue of IJPR is dedicated to Prof. Hossein-zadeh, Mashhad, Iran, for his lifetime achievement. I would like to take this opportunity to thank all reviewers of this special issue for their commitment and hard work. Without their dedication and tremendous efforts, this issue would not have been possible.

It is hoped that these papers can document an increase in the Iranian traditional medicine and lead to the development of new drugs effective in the treatment of diseases.

*Faraz Mojab is currently working as a professor in Department of Pharmacognosy, School of the Pharmacy, Shaheed Beheshti University of Medical Sciences, Tehran. He can be reached at sfmojab@sbmu.ac.ir*

